# Pseudophakic corneal edema caused by Descemet membrane detachment using high-resolution swept-source OCT imaging

**DOI:** 10.3205/oc000244

**Published:** 2024-09-23

**Authors:** Maximilian K. Köppe, Ramin Khoramnia, Gerd U. Auffarth, Victor A. Augustin

**Affiliations:** 1 University Eye Clinic Heidelberg, International Vision Correction Research Centre (IVCRC), Heidelberg, Germany

**Keywords:** cataract surgery, Descemet membrane detachment, corneal edema, case report

## Abstract

**Background:**

Small Descemet membrane detachments after cataract surgery are relatively common and most cases do not require any secondary surgical intervention and can be treated conservatively. However, in case of advanced Descemet membrane detachment (DMD), it needs to be recognized and treated appropriately. The advent of anterior segment imaging using optical coherence tomography (OCT) technology has made diagnosing pathologies of the anterior segment accurate and time efficient and has proven as an invaluable tool to guide decision making.

**Case presentation:**

A 71-year-old patient presented after complicated cataract surgery with decreased visual acuity and cloudy vision. On examination, best corrected visual acuity was 1.5 logMAR. A high-resolution swept-source OCT (Anterion, Heidelberg Engineering, Heidelberg, Germany) was used to better evaluate and visualize the extent of DMD. An anterior chamber gas bubble was injected to reattach the Descemet membrane (DM) to the corneal stroma. The success of the surgery was visualized using the high-resolution swept-source OCT. This revealed a completely attached Descemet membrane.

**Conclusions:**

Clinically, it can be difficult to distinguish the etiology of epithelial and stromal edema post cataract surgery. This case demonstrated the clinical usefulness using high resolution swept source imaging to guide clinical decision making in evaluating timing and treatment success of pneumodescemetopexy after complicated cataract surgery.

## Background

Clinically significant Descemet membrane detachment (DMD) with decreased visual acuity is relatively rare with a reported incidence of 0.044% after cataract surgery [1]. Visualizing DMD in specular microscopy can be difficult as it may only be a small detachment or examination is obscured by corneal edema. High resolution swept source optical coherence tomography (OCT) can serve as a quick and accurate diagnostic feature. DMD usually occurs during cataract surgery when aqueous enters the corneal stroma-Descemet membrane interface created by the corneal incisions. This can occur, for example, during irrigation-aspiration or stromal hydration [1]. Ti and colleagues found preexisting endothelial disease as the only risk factor for DMD after cataract surgery [2].

For minor DMD conservative treatment including observation, topical steroids and hyperosmolar solutions can be an option [3]. For larger DMD, such as in this case, pneumodescemetopexy with intracameral air or gas is the first surgical option mainly due to its ease of execution, rapid visual recovery and good safety profile [2]. 

## Case description

**Figure 1:**
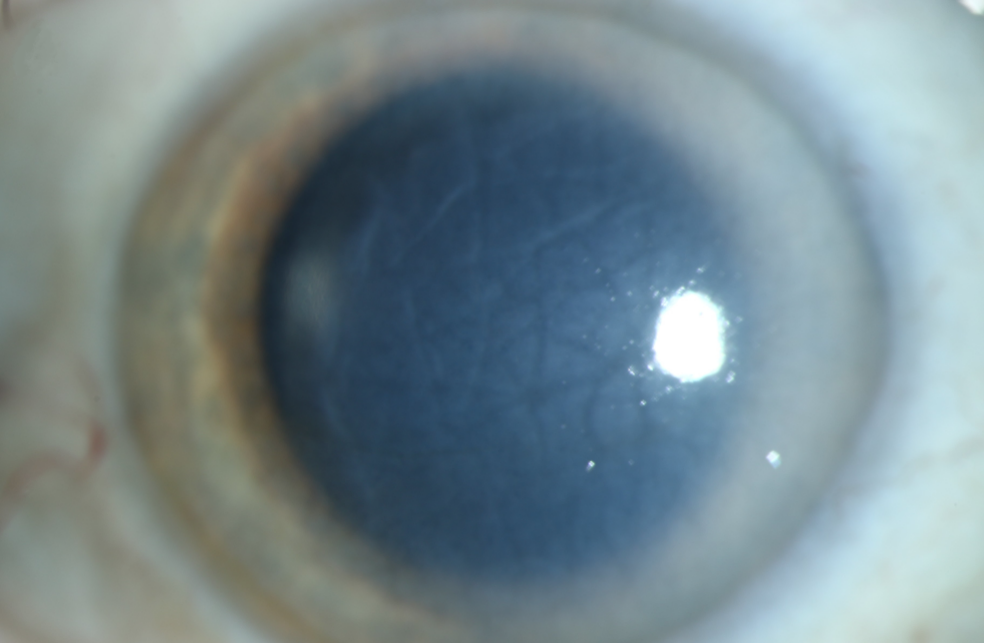
Slit-lamp image of the corneal edema

**Figure 2:**
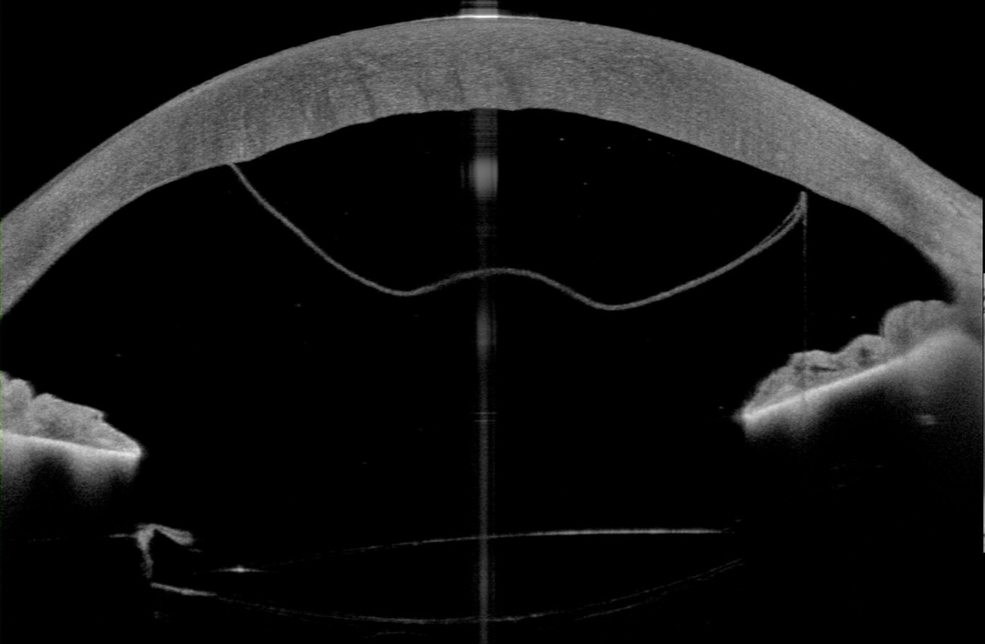
High-resolution anterior segment OCT showing a significantly centrally detached Descemet membrane towards the anterior chamber

**Figure 3:**
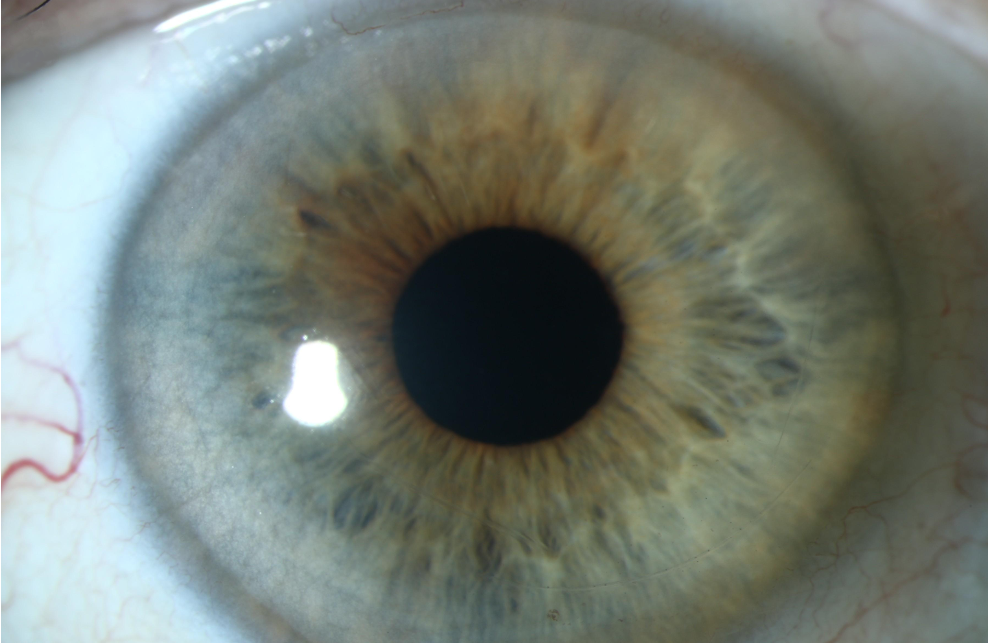
Slit-lamp image of the clear central cornea with no edema after surgery

**Figure 4:**
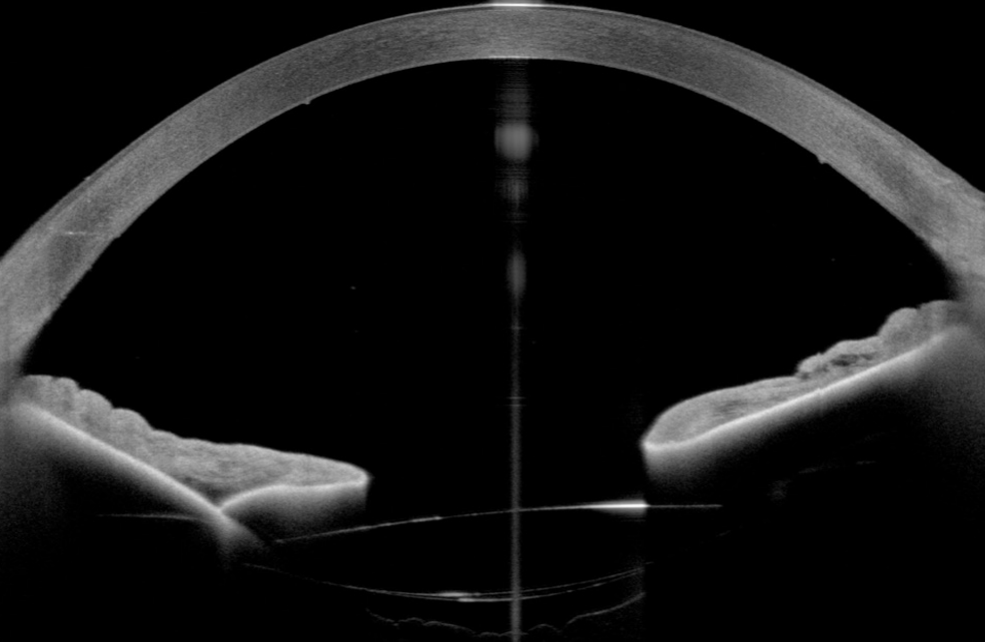
High-resolution anterior segment OCT showing a fully attached Descemet membrane

A 71-year-old patient presented after complicated cataract surgery with decreased visual acuity and cloudy vision. On examination, best corrected visual acuity was 1.5 logMAR. Slit-lamp examination revealed diffusely epithelial and stromal edema with Descemet folds centrally and DMD. A high-resolution swept-source OCT (Anterion, Heidelberg Engineering, Heidelberg, Germany) was used to better evaluate and visualize the extent of DMD. Figure 1 (Fig. 1) shows a slit-lamp image of the eye and Figure 2 (Fig. 2) shows the anterior segment OCT. The OCT shows a significantly centrally detached Descemet membrane (DM) towards the anterior chamber. Pre-operatively endothelial cell count could not be measured due to significant corneal edema. We decided to perform a complete anterior chamber air tamponade under topical anesthesia and eventually reduced the air bubble to 80% anterior chamber volume after one hour. On the first day postoperatively, central corneal edema decreased, visual acuity improved to 0.30 logMAR and anterior segment OCT showed a fully attached Descemet membrane. Mean postoperative endothelial cell count after day 1 was 1,149 cells/mm^2^. After 3 months visual acuity further improved to 0.1 logMAR, with no central corneal edema (Figure 3 (Fig. 3)) and normal intraocular pressure. The OCT shows a fully attached DM (Figure 4 (Fig. 4)).

## Discussion and conclusions

Corneal edema after cataract surgery is a very common finding and is mostly self-limiting. However, in terms of persistent corneal edema it can be difficult to clinically distinguish the etiology of epithelial and stromal edema after cataract surgery. Even though DMD being a relatively rare postoperative complication after cataract surgery [1], this case report showed that it can cause significant reduction in visual acuity. Ti and colleagues suggested pneumodescemetopexy as the treatment of choice for large DMD [2]. However, visualizing and evaluating the extent of DMD using specular microscopy as well as guiding surgical decision making can be very challenging.

This case demonstrated that high resolution swept source OCT serves as a quick and invaluable tool to facilitate visualizing the extent of DMD and to guide clinical decision making in evaluating timing and treatment success of pneumodescemetopexy after cataract surgery. This case also confirmed that large DMD can successfully be treated using anterior chamber air injection.

## Notes

### Availability of data and materials

Data and materials are on file at University Eye Clinic Heidelberg, Germany.

### Consent for publication

Written informed consent was obtained from the patient for publication of this case report and any accompanying images.

### Competing interests

The authors declare that they have no competing interests.
